# Comparison of atrial fibrillation prevalence and in-hospital cardiovascular outcomes between patients undergoing allogeneic versus autologous hematopoietic stem cell transplantation: insights from the national inpatient sample

**DOI:** 10.1038/s41598-024-65294-9

**Published:** 2024-07-22

**Authors:** Satyam Krishan, Zain Ul Abideen Asad, Dionisia Quiroga, Sanam M. Ghazi, Cooper Quartermaine, Zachary Braunstein, Onaopepo Kola-Kehinde, Adnan Shaaban, Alma Habib, Sarah Khan, Richard Cheng, Jonathan E. Brammer, Daniel Addison

**Affiliations:** 1 Department of Medicine, The University of Oklahoma Health Sciences Center, Oklahoma City, OK USA; 2 Division of Medical Oncology, The Ohio State University Medical Center, Columbus, OH USA; 3 Cardio-Oncology Program, Division of Cardiology, Davis Heart and Lung Research Institute, The Ohio State University Medical Center, 473 West 12th Avenue, Columbus, OH USA; 4 Division of Hospital Medicine, The Ohio State University Medical Center, Columbus, OH USA; 5 Division of Hematology, The Ohio State University Medical Center, Columbus, OH USA; 6 Cardio-Oncology Program, Division of Cardiology, University of Washington Medical Center, Seattle, WA USA; 7 Division of Cancer Prevention and Control, The Ohio State University, Columbus, OH USA

**Keywords:** Bone marrow transplantation, Hematopoietic stem cell transplantation, Allogeneic, Autologous, Atrial fibrillation, Arrhythmias, Cardiovascular outcomes, Mortality, Cardiology, Medical research, Oncology

## Abstract

Hematopoietic stem cell transplantation (HSCT) is a potentially curative therapy for several malignant and non-malignant hematologic conditions. However, patients undergoing HSCT are at increased risk of developing serious cardiovascular events. Whether cardiovascular risks differ by the type of transplantation strategy used, allogeneic versus autologous HSCT, is unknown. Leveraging the National Inpatient Sample (2016–2019), we assessed the incidence of early cardiovascular events by HSCT mode (allogeneic vs autologous). The primary outcome was the incidence of atrial fibrillation (AF). The secondary outcome was the occurrence of any major adverse cardiac events (MACE), defined as acute heart failure, myocardial infarction (MI), symptomatic atrial or ventricular arrhythmia or heart block, and cardiovascular death. Outcomes were compared between those undergoing allogeneic versus autologous HSCT. Multivariable regression, adjusting for cardiovascular and cancer-related factors, was used to define the association between pre-HSCT factors and MACE. We further assessed the effect of acute cardiovascular events on in-patient mortality by calculating adjusted odds ratio (aOR) with corresponding 95% confidence intervals (CI) and *p*-values. Overall, 64,705 weighted hospitalizations for HSCT were identified, of which 22,655 (35.0%) were allogeneic HSCT and 42,050 (65.0%) were autologous HSCT. The prevalence of AF was 9.1%, and 12.1% for any arrhythmia. In multivariable regression, allogeneic HSCT was associated with higher adjusted odds of peri-HSCT acute heart failure (aOR 2.64; 1.86–3.76; *p* < 0.0001), QT prolongation (aOR 1.40; 1.04–1.88; *p* = 0.025), MI (aOR 2.87; 1.16–7.11; *p* = 0.023), any major cardiovascular complication (aOR 1.16; 1.03–1.32; *p* = 0.016), and inpatient mortality (aOR 4.87; 3.60–6.58; *p* < 0.0001). Following cerebrovascular events, AF was the strongest predictor of mortality. Allogeneic HSCT was associated with higher odds of in-hospital cardiovascular complications among patients undergoing HSCT.

## Introduction

Nearly 100,000 people are diagnosed with aggressive leukemias and lymphomas every year in the United States^1^. Hematopoietic stem cell transplantation (HSCT) has emerged as a potentially curative therapy for patients with these aggressive hematologic malignancies^2,3^. HSCT dramatically improves the survival for malignancies and it is associated with up to > 90% 5-year survival in certain conditions^4^. However, HSCT is also associated with an increased risk of cardiovascular complications, including atrial fibrillation (AF), heart failure (HF), and other serious cardiovascular events^5–7^. Although there is considerable variation in published literature about the true incidence of these major cardiovascular complications, the comparative effect of different types of HSCT treatments and the risk of developing these cardiovascular complications is unclear.

Among other cardiovascular complications, HSCT appears to be associated with an increase in the risk of developing symptomatic AF^5^. AF and HF are the most common adverse cardiovascular events following HSCT and have been associated with adverse outcomes and increased risk of long-term mortality in these patients^5,8,9^. Our objective was to assess the incidence and predictors of developing adverse cardiovascular outcomes stratified by the allogenic vs autologous HSCT using a large nationally representative database.

## Methods

### Data source

From the National Inpatient Sample (NIS), we identified all patients treated with HSCT from 2016 to 2019 for any malignant condition. Hospital admissions before 2016 were excluded as the International Classification of Diseases, 10th Revision, Clinical Modification (ICD-10-CM) coding system came into effect in 2015. NIS is part of a family of databases developed under the Healthcare Cost and Utilization Project (HCUP) through a Federal-State-Industry Partnership and sponsored by the Agency for Healthcare Research and Quality (AHRQ) and is the largest publicly available inpatient healthcare database. NIS contains data on approximately 7 million unweighted and 35 million weighted hospitalizations each year which can be used to compute national estimates of healthcare utilization, costs, and outcomes^10^. Due to the de-identified nature of NIS, the need for informed consent and Institutional Review Board (IRB) approval was waived. The NIS adheres to the 2013 Declaration of Helsinki for the conduct of human research.

### Study population

Patients undergoing HSCT were identified using International Classification of Diseases, 10th Revision, Procedure Coding System (ICD-10-PCS) codes “30243C0”, “30243G0”, “30243X0”, “30243Y0”, “30233C0”, “30233G0”, “30233X0” and “30233Y0” for autologous HSCT and “30243G2”, “30243G3”, “30243G4”, “30243U-”, “30243X2”, “30243X3”, “30243X4”, “30243Y2”, “30243Y3”, “30243Y4”, “30233G2”, “30233G3”, “30233G4”, “30233U”, “30233X2”, “30233X3”, “30233X4”, “30233Y2”, “30233Y3” and “30233Y4” for allogeneic HSCT. Patients aged < 18 years were excluded from this analysis.

Clinical and demographic characteristics and inpatient cardiovascular outcomes (AF, atrial flutter [AFL], supraventricular tachycardia [SVT], QT prolongation, ventricular tachycardia [VT], ventricular fibrillation [VF], first-degree atrioventricular [AV] block, second-degree AV block, third-degree AV block, sick sinus syndrome [SSS], cardiac arrest, acute heart failure [HF] exacerbation, cardiogenic shock, stroke, transient ischemic attack [TIA], type 2 myocardial infarction [MI], non-ST elevation MI [NSTEMI], ST-elevation MI [STEMI]), pericarditis and pericardial effusion were compared between patients undergoing allogeneic and autologous HSCT. The primary outcome was the incidence of atrial fibrillation (AF). The secondary outcome was the occurrence of any major adverse cardiac events (MACE), defined as acute heart failure, myocardial infarction (MI), symptomatic atrial or ventricular arrhythmia or heart block, and cardiovascular death. Data supporting the findings of this study are available from authors upon reasonable request.

### Statistical analyses

Descriptive statistics are presented as frequencies and percentages with corresponding 95% confidence interval (CI) for categorical variables and as mean with corresponding 95% CI for continuous variables. Baseline characteristics and unadjusted outcomes were compared using the Pearson χ^2^ test and univariable logistic regression for categorical variables and univariable linear regression for continuous variables. For assessment of the independent association of type of HSCT with outcomes, a multivariable logistic regression model was utilized to calculate the adjusted odds ratio (aOR) with corresponding 95% CI and *p*-value. The multivariable regression model adjusted for demographics including age, sex, race/ethnicity, insurance status, median household income quartile, and underlying comorbidities, including hypertension, complicated and uncomplicated diabetes mellitus (DM), coronary artery disease (CAD), dyslipidemia, peripheral arterial disease (PAD), chronic HF, chronic obstructive pulmonary disease (COPD), chronic kidney disease (CKD), cerebrovascular disease, obesity, cirrhosis, anemia, thyroid dysfunction, tobacco use, alcohol use, substance use, hematologic malignancies (including Hodgkin’s lymphoma, non-Hodgkin’s lymphoma, multiple myeloma, acute and chronic myeloid leukemia, acute and chronic lymphoid leukemia, monocytic leukemia), and metastatic solid tumor disease. A *p*-value of < 0.05 was considered statistically significant. All statistical analyses were performed using the software Stata/BE version 17.0 (StataCorp).

### Ethical approval

This material is the authors’ original work and has not been previously published elsewhere. The paper is not currently being considered for publication elsewhere. The paper reflects the authors’ own research and analysis in a truthful and complete manner. All authors have been personally and actively involved in substantial work leading to the paper and will take responsibility for its content. The NIS adheres to the 2013 Declaration of Helsinki for the conduct of human research. Due to the de-identified nature of the NIS database, the need for Institutional Review Board (IRB) approval was waived.

### Informed consent

Due to the de-identified nature of the NIS database, the need for informed consent was waived.

## Results

Overall, 64,705 weighted hospitalizations of patients undergoing HSCT were identified. Among these, 22,655 (35.0%) underwent allogeneic HSCT, and 42,050 (65.0%) underwent autologous HSCT. The mean age was 55.6 years, 41.3% were females, 67.9% were White, 12.5% were Black, 10.5% Hispanic, 3.2% Asian, and 0.3% were Native American.

Patients undergoing allogeneic compared to autologous HSCT were younger (average age 52.3 years vs 57.3 years, *p* < 0.001), and more likely to be female 43.8% vs 40.0%, *p* < 0.001), White (70.4% vs 66.6%, *p* < 0.001), Hispanic (11.5% vs 10.0%, *p* < 0.001) or Asian (4.2% vs 2.6%, *p* < 0.001), but less likely to be Black (7.8% vs 15.1%, *p* < 0.001). Allogeneic HSCT patients had a higher burden of comorbidities including cerebrovascular disease (1.8% vs 1.0%, *p* < 0.001), cirrhosis (0.5% vs 0.2%, *p* = 0.02), and coagulopathy (16.5% vs 14.2%, *p* = 0.003) but lower DM (13.9% vs 15.8%, *p* = 0.003), dyslipidemia (19.9% vs 23.4%; *p* < 0.001), CKD (4.9% vs 12.2%, *p* < 0.001), tobacco use (4.7% vs 6.4%, *p* < 0.001) and substance abuse (5.9% vs 7.6%, *p* < 0.001) compared to those undergoing autologous HSCT. Information on other clinical and demographic characteristics is available in Table 1. Information on indications for HSCT based on HSCT type is available in Data Supplement.

**Table 1:** Clinical and demographic characteristics of patients undergoing HSCT stratified by the type of HSCT status.

	Allogeneic (n = 22,655)	Autologous (n = 42,050)	*p*-value
Mean age	52.3	57.3	< 0.0001
Female (%)	9910 (43.8%)	16,815 (40.0%)	0.0003
Race (%)			< 0.0001
White	15,450 (70.4%)	26,850 (66.6%)	
Black	1705 (7.8%)	6065 (15.1%)	
Hispanic	2525 (11.5%)	4030 (10%)	
Asian or Pacific Islander	930 (4.2%)	1045 (2.6%)	
Native American	100 (0.5%)	110 (0.3%)	
Other	1240 (5.7%)	2205 (5.5%)	
Insurance status (%)			< 0.0001
Medicare	5350 (24.7%)	12,780 (31.4%)	
Medicaid	2720 (12.6%)	4135 (10.2%)	
Private insurance	13,305 (61.5%)	23,245 (57.2%)	
Self-pay	260 (1.2%)	505 (1.2%)	
Median household income quartile (%)			0.0011
0–25th percentile	4065 (18.3%)	8570 (20.7%)	
26–50th percentile (median)	5035 (22.6%)	9915 (23.9%)	
51st–75th percentile	6065 (27.3%)	11,040 (26.7%)	
76–100th percentile	7095 (31.9%)	11,905 (28.7%)	
Hypertension (%)	10,445 (46.1%)	19,955 (47.5%)	0.17
Diabetes (%)	3160 (13.9%)	6660 (15.8%)	0.0027
Chronic HF (%)	775 (3.4%)	1520 (3.6%)	0.56
CAD (%)	1615 (7.1%)	2890 (6.9%)	0.59
PAD (%)	220 (1.0%)	470 (1.1%)	0.48
Dyslipidemia (%)	4505 (19.9%)	9850 (23.4%)	< 0.0001
COPD (%)	210 (0.9%)	290 (0.7%)	0.13
CKD (%)	1115 (4.9%)	5115 (12.2%)	< 0.0001
CVD (%)	400 (1.8%)	415 (1.0%)	0.0001
Obesity (%)	2140 (9.5%)	3615 (8.6%)	0.14
Cirrhosis (%)	110 (0.5%)	100 (0.2%)	0.02
Anemia (%)	18,970 (83.7%)	34,815 (82.8%)	0.37
Thyroid dysfunction (%)	2590 (11.4%)	4495 (10.7%)	0.18
Coagulopathy (%)	3735 (16.5%)	5975 (14.2%)	0.0032
Alcohol use (%)	180 (0.8%)	280 (0.7%)	0.39
Tobacco use (%)	1070 (4.7%)	2715 (6.4%)	0.0001
Substance abuse (%)	1345 (5.9%)	3200 (7.6%)	0.0007

For n < 11, the absolute numbers are not reported as per Healthcare Cost and Utilization Project recommendations.

*CAD* coronary artery disease, *CKD* chronic kidney disease, *COPD* chronic obstructive pulmonary disease, *CVD* cerebrovascular disease, *HF* heart failure, *HSCT* hematopoietic stem cell transplantation, *PAD* peripheral artery disease.

### Comparison of in-hospital cardiovascular outcomes

The incidence of in-hospital cardiovascular events was 13.0%, with the most common being arrhythmias (12.1%), followed by acute HF exacerbation (1.2%). Allogeneic HSCT recipients had a higher incidence of inpatient mortality (4.6% vs 1.0%, *p* < 0.001), acute HF exacerbation (1.9% vs 0.9%, *p* < 0.001), cardiogenic shock (0.4% vs 0.2%, *p* = 0.033), type 2 MI (0.6% vs 0.2%, *p* < 0.001), pericardial effusions (2.2% vs 0.5%, *p* < 0.001), and pericarditis (0.3% vs 0.1%, *p* = 0.006) compared to autologous HSCT recipients on a crude analysis. The distribution of other relevant outcomes is shown in Table 2.

**Table 2:** Crude in-hospital outcomes for patients undergoing HSCT stratified by the type of HSCT status.

	Allogeneic (n = 22,655)	Autologous (n = 42,050)	*p*-value
Atrial fibrillation (%)	1905 (8.4%)	4005 (9.5%)	0.036
Atrial flutter (%)	320 (1.4%)	830 (2.0%)	0.02
Supraventricular tachycardia (%)	485 (2.1%)	900 (2.1%)	1.00
QT prolongation (%)	515 (2.3%)	680 (1.6%)	0.013
Ventricular tachycardia (%)	275 (1.2%)	445 (1.1%)	0.43
Ventricular fibrillation (%)	< 11	30 (0.07%)	0.25
1st degree AV block (%)	110 (0.5%)	260 (0.6%)	0.34
2nd degree AV block (%)	30 (0.1%)	20 (0.05%)	0.095
3rd degree AV block (%)	20 (0.09%)	20 (0.05%)	0.37
Sick sinus syndrome (%)	25 (0.11%)	40 (0.09%)	0.81
Any arrhythmia	2610 (11.5%)	5190 (12.4%)	0.18
Cardiac arrest (%)	105 (0.5%)	200 (0.5%)	0.93
Acute HF exacerbation (%)	430 (1.9%)	360 (0.9%)	< 0.0001
Cardiogenic shock (%)	85 (0.4%)	70 (0.2%)	0.033
Pericarditis	65 (0.3%)	40 (0.1%)	0.006
Pericardial effusion	495 (2.2%)	225 (0.5%)	< 0.0001
Type 2 MI	125 (0.6%)	80 (0.2%)	0.0004
NSTEMI	70 (0.3%)	75 (0.2%)	0.14
STEMI	35 (0.2%)	25 (0.06%)	0.09
MACE	2935 (13.0%)	5475 (13.02%)	0.91
Inpatient mortality (%)	1030 (4.6%)	430 (1.0%)	< 0.0001
Discharge disposition			< 0.0001
Routine	15,560 (68.7%)	35,245 (83.8%)	
Short-term hospital	60 (0.3%)	90 (0.2%)	
Another type of facility	500 (2.2%)	900 (2.1%)	
Home health care (HHC)	5505 (24.3%)	5330 (12.7%)	
Against medical advice (AMA)	< 11	40 (0.09%)	

For n < 11, the absolute numbers are not reported as per Healthcare Cost and Utilization Project recommendations.

*AV* atrioventricular, *HF* heart failure, *HSCT* hematopoietic stem cell transplantation, *MACE* major adverse cardiovascular event, *NSTEMI* non-ST-elevation myocardial infarction, *STEMI* ST-elevation myocardial infarction; *type 2 MI* type 2 myocardial infarction.

**Figure 1:**
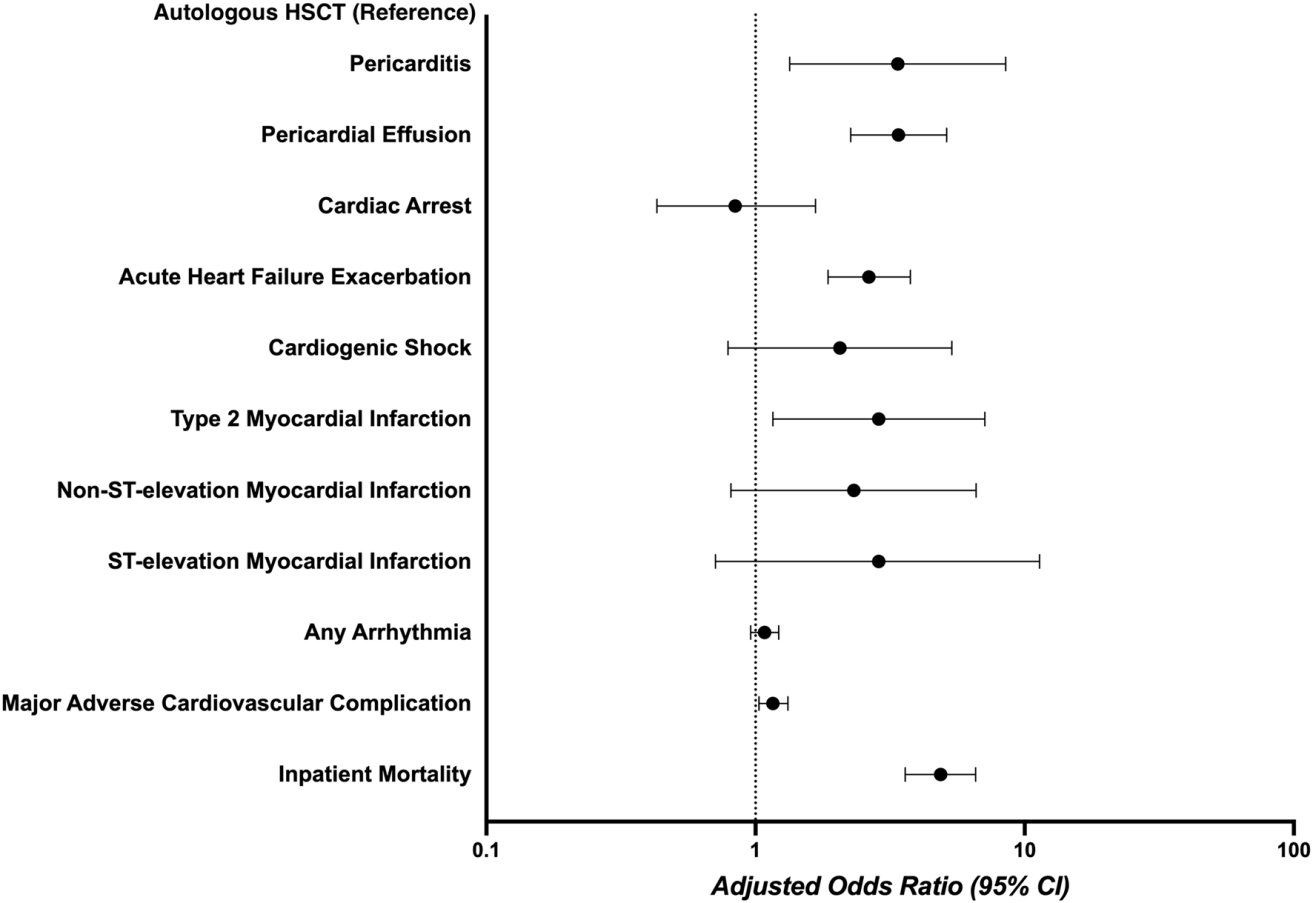
Forest plot showing a comparison of cardiovascular outcomes including pericarditis, pericardial effusion, cardiac arrest, acute HF exacerbation, cardiogenic shock, type 2 myocardial infarction (MI), Non-ST-elevation MI, ST-elevation MI, any arrhythmia, major adverse cardiovascular complication and inpatient mortality between allogenic HSCT and autologous HSCT recipients after adjusting for demographic data including age, sex, race, insurance status, and median household income, comorbidities listed in Table 1 and underlying cancer type.

To assess the independent association of HSCT strategy with adverse outcomes, we used multivariable regression models, adjusted for potential confounders such as age, sex, race, insurance status, income quartile, CAD, PAD, chronic HF, cerebrovascular disease, CKD, anemia, coagulopathy, cirrhosis, thyroid dysfunction, and type of hematologic malignancy. On the adjusted analysis, allogeneic HSCT was found to be associated with higher odds of inpatient mortality (aOR 4.87; 95% CI 3.60–6.58; *p* < 0.0001), acute HF exacerbation (aOR 2.64; 95% CI 1.86–3.76; *p* < 0.0001), type 2 MI (aOR 2.87; 95% CI 1.16–7.11; *p* = 0.023), pericardial effusion (aOR 3.40; 95% CI 2.26–5.13; *p* < 0.001), pericarditis (aOR 3.38; 95% CI 1.34–8.51; *p* = 0.01) and any major adverse cardiovascular complication (aOR 1.16; 95% CI 1.03–1.32; *p* = 0.016). Figure 1 shows a comparison of cardiovascular outcomes between the allogeneic and autologous HSCT recipients.

### Comparative effect on AF and other serious arrhythmia risk

**Figure 2:**
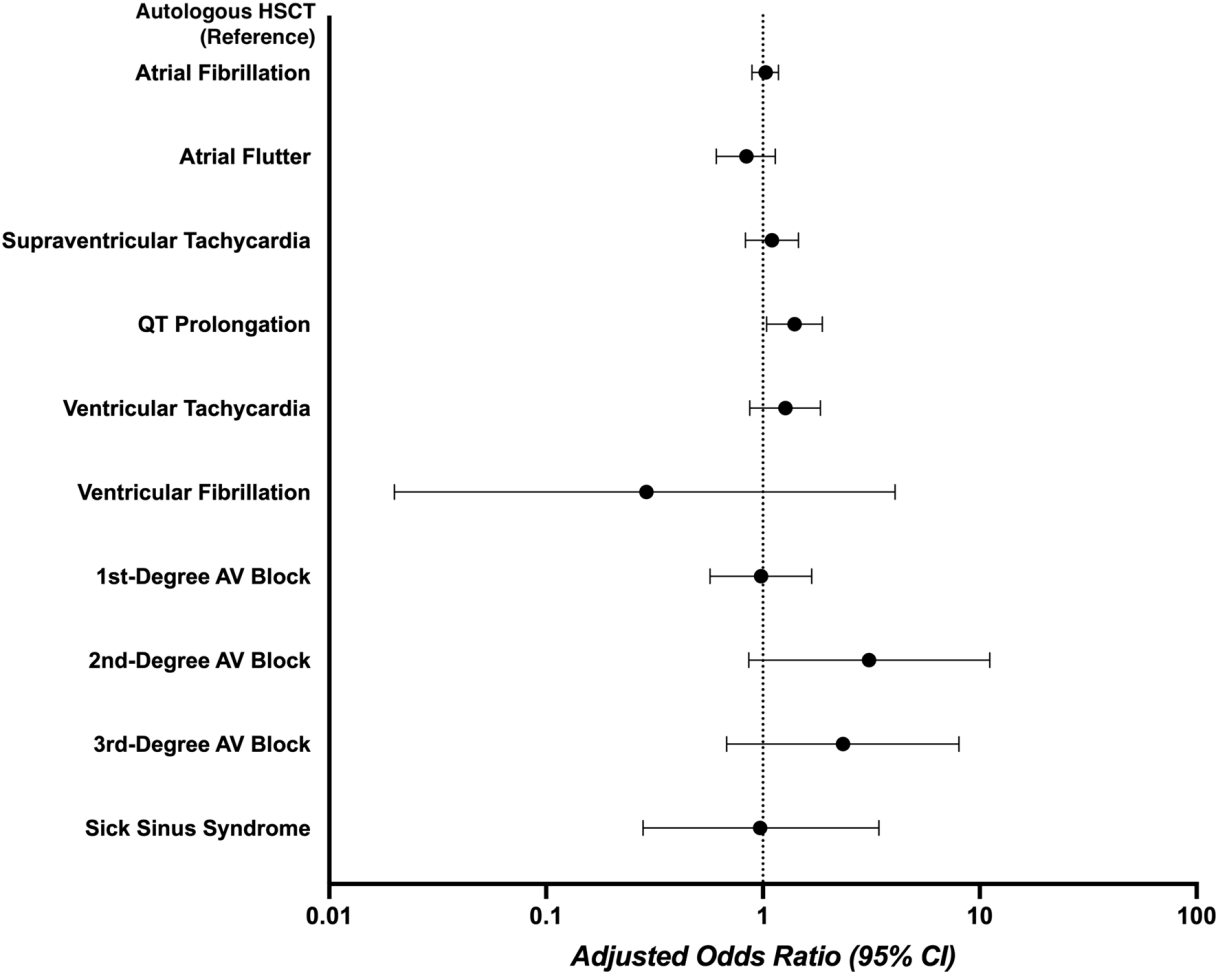
Forest plot showing a comparison of all arrhythmias including atrial fibrillation, atrial flutter, supraventricular tachycardia, QT prolongation, ventricular tachycardia, ventricular fibrillation, 1st-degree AV block, 2nd-degree AV block, 3rd-degree AV block and sick sinus syndrome between allogenic HSCT and autologous HSCT recipients after adjusting for demographic data including age, sex, race, insurance status, and median household income, and underlying comorbidities listed in Table 1.

The overall burden of arrhythmias in patients undergoing HSCT patients was 12.1% and the most prevalent arrhythmia was AF (9.1%) followed by SVT (2.1%) and AFL (1.8%). Patients undergoing allogeneic HSCT had higher rates of QT prolongation (2.3% vs 1.6%, *p* = 0.013), but lower AF (8.4% vs 9.5%, *p* = 0.036) and AFL (1.4% vs 2.0%; *p* = 0.02) compared to those undergoing autologous HSCT on a crude analysis (Table 2). Results demonstrating a comparison of arrhythmia outcomes are shown in Fig. 2. In multivariable analysis, allogeneic HSCT was associated with higher odds of QT prolongation (aOR 1.40; 95% CI 1.04–1.88; *p* = 0.025); this association was independent of age, sex, race, insurance status, income quartile, and important comorbidities such as underlying CAD, chronic HF, hypertension, anemia, and coagulopathy on a multivariable logistic regression analysis. There was no difference in AF, AFL, SVT, VT, VF, first-degree AV block, second-degree AV block, third-degree AV block, SSS or cumulative arrhythmia prevalence. There was a trend towards higher odds of second-degree AV block (aOR 3.09; 95% CI 0.86–11.12; *p* = 0.084) with allogeneic HSCT.

### Comparative effect of cardiovascular events on inpatient mortality

**Figure 3:**
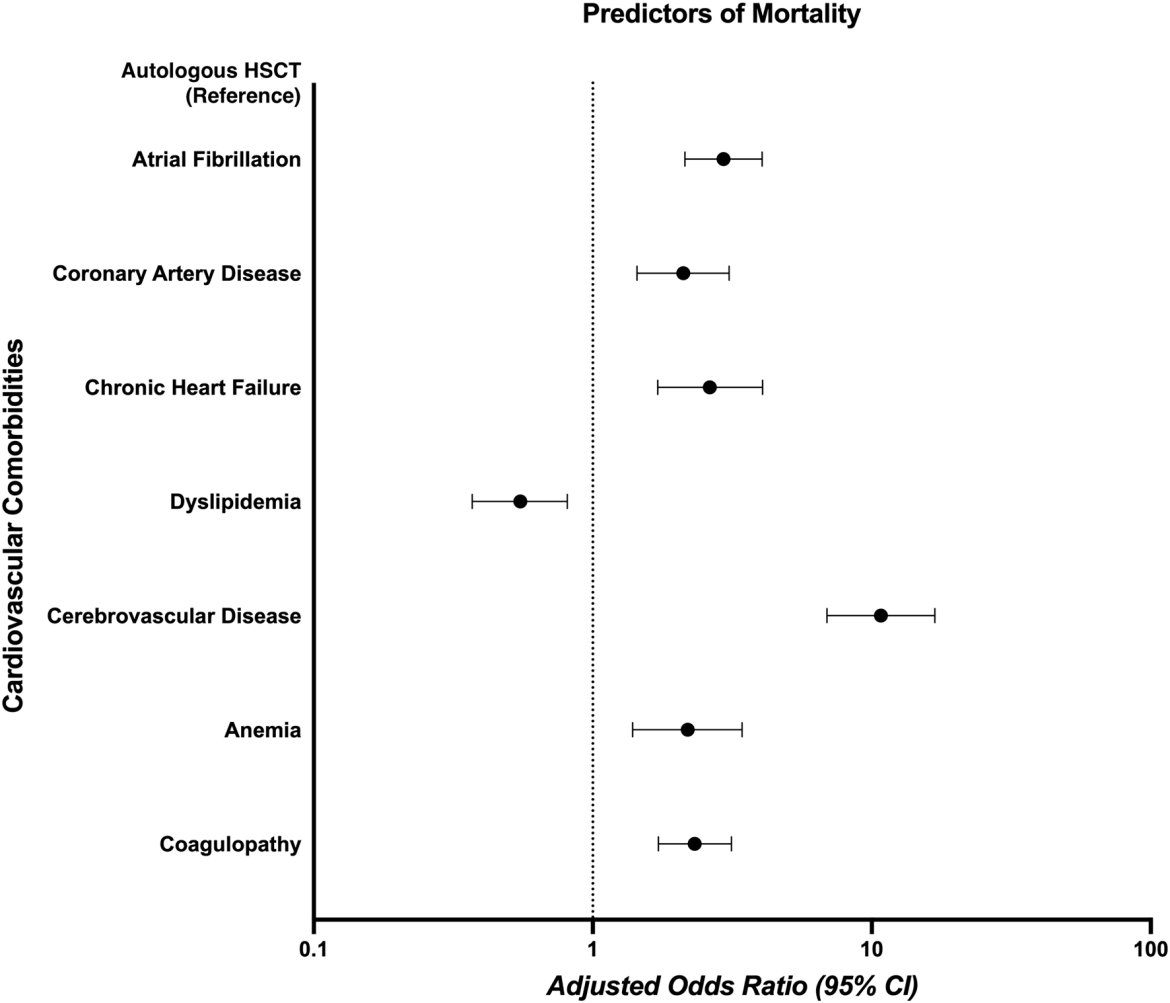
Forest plot showing significant cardiovascular predictors of inpatient mortality in all HSCT recipients.

The overall mortality rate among all HSCT recipients was 2.3%. Furthermore, the cardiovascular predictors of inpatient mortality among all HSCT recipients included AF (aOR 2.94; 95% CI 2.14–4.05; *p* < 0.001), CAD (aOR 2.11; 95% CI 1.44–3.08; *p* < 0.001), chronic HF (aOR 2.63; 95% CI 1.71–4.05; *p* < 0.001), dyslipidemia (aOR 0.55; 95% CI 0.37–0.81; *p* = 0.002), cerebrovascular disease (aOR 10.79; 95% CI 6.91–16.85; *p* < 0.001), anemia (aOR 2.19; 95% CI 1.39–3.43; *p* = 0.001) and coagulopathy (aOR 2.32; 95% CI 1.72–3.14) (Fig. 3). Cardiovascular predictors of mortality stratified based on HSCT type are available in Data Supplement.

## Discussion

In this large and nationally representative sample of HSCT recipients, we have shown that:

1. The overall arrhythmia burden in these patients is high (12.1%), with the most prevalent arrhythmia being AF (9.1%) followed by SVT (2.1%) and AFL (1.8%).
2. Allogeneic HSCT saw higher odds of QT prolongation. This association remained, even after accounting for age, gender, race, socioeconomic factors, and important comorbidities such as hypertension, DM, chronic HF, CAD, PAD, CKD, and obesity.
3. Allogeneic HSCT was associated with a greater than twofold relative increase in inpatient mortality, acute HF exacerbation, MI, pericarditis, and pericardial effusion, and an increase in the composite risk of any major cardiovascular complication.
4. Cardiovascular predictors of inpatient mortality among HSCT recipients included AF, CAD, chronic HF, dyslipidemia, cerebrovascular disease, anemia, and coagulopathy.

**Figure 4:**
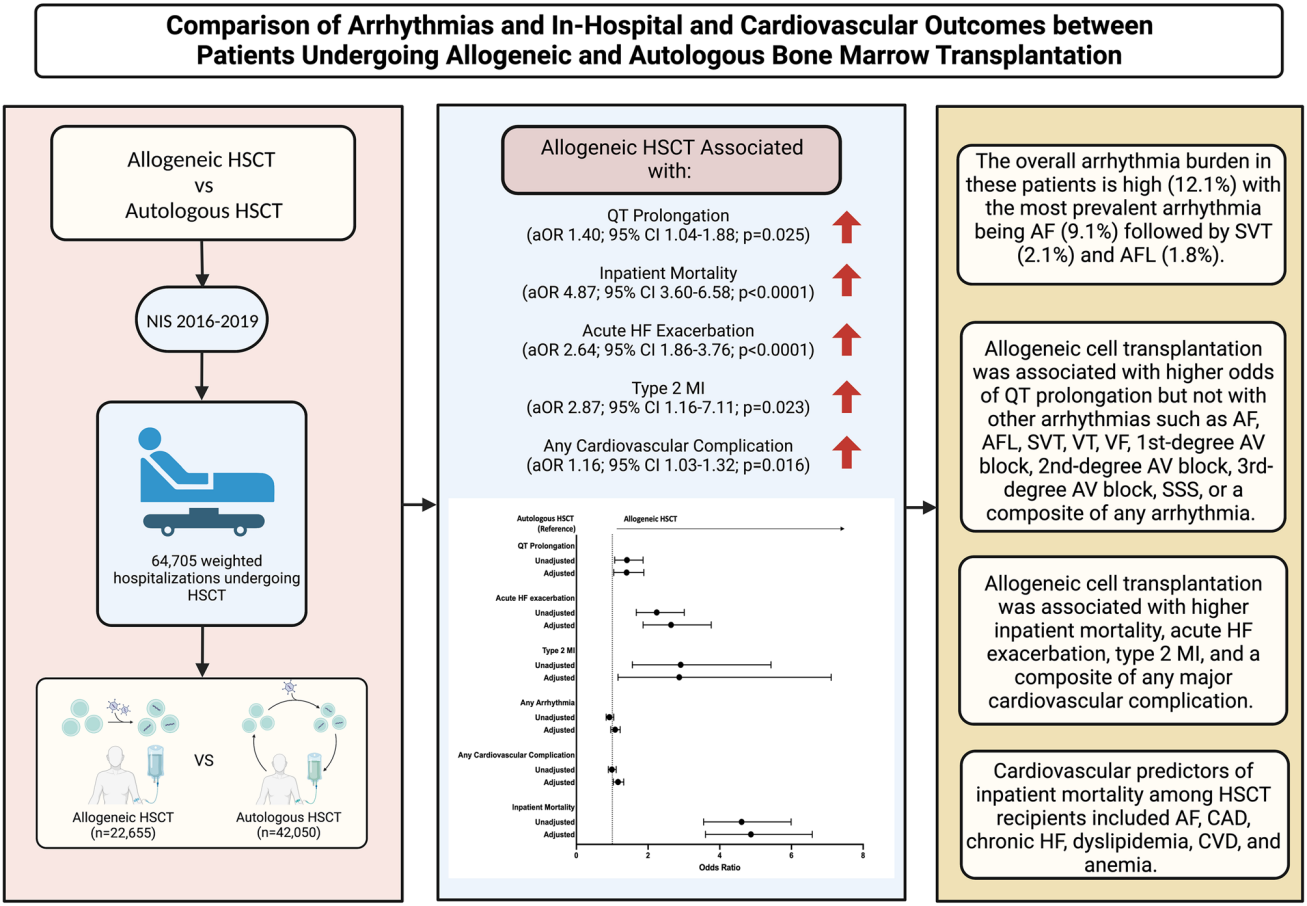
Central Illustration depicting study design; *AF* atrial fibrillation, *AFL* atrial flutter, *AV Block* atrioventricular block, *CAD* coronary artery disease, *CVD* cerebrovascular disease, *HF* heart failure, *HSCT* hematopoietic stem cell transplantation, *MI* myocardial infarction, *SSS* sick sinus syndrome, *SVT* supraventricular tachycardia, *VF* ventricular fibrillation, *VT* ventricular tachycardia.

The Central Illustration (Fig. 4) shows an overview of the study design and results. The observation of increased cardiovascular event risk with HSCT, particularly allogeneic transplantation, adds to a growing body of literature. In smaller retrospective investigations, the increased burden of arrhythmia, particularly AF, has approached 20% in long-term follow-up^11^. Although a higher crude prevalence of AF and AFL was seen in the autologous HSCT group, upon adjustment for confounders, autologous HSCT was not found to be independently associated with higher odds of AF or AFL compared to the allogeneic HSCT group. Regarding arrhythmias, QT prolongation was the primary difference seen between the two populations, upon adjustment for confounders. The association of allogeneic HSCT with a higher prevalence of QT prolongation in comparison to autologous HCT could potentially be explained by differences in chemotherapeutic agents used for the underlying disease (e.g. cardiotoxic anthracyclines for AML), the potential need for prolonged antifungal and antibacterial prophylaxis, additional supportive measures including anti-emetics or a combination of these variables^12–15^. Allogeneic HSCT frequently requires immunomodulatory regimens to achieve myeloablation and minimize the risk of immunologic complications^16^. Notably, patients undergoing allogeneic HSCT were more likely to receive cytotoxic therapies which may contribute to cardiotoxicity^17^. Further, the engraftment of stem cells themselves may enhance the innate immune response, allowing a fertile state for enhanced arrhythmogenesis and atherosclerosis development, a condition more prominent than seen with autologous HSCT^18^. Although there has been a trend toward the avoidance of potentially cytotoxic agents in recent years, the presence of factors such as immunomodulation, immune activation following HSCT, and graft-versus-host effects may potentially enhance cardiovascular risk, even within months of HSCT^19^. Additionally, drugs such as cyclophosphamide used for graft-versus-host disease prophylaxis, have been associated with an increased risk for early (within 180 days post-transplantation) cardiovascular complications such as heart failure, pericarditis, acute coronary syndrome, and arrhythmia^20,21^. Allogeneic HSCT was also associated with higher odds of inpatient mortality and cardiovascular complications such as acute HF exacerbation and type 2 MI which could be attributed to the above-mentioned factors. Notably, these observations are compounded by the disparately increased longer-term risk of cardiovascular disease seen even within years of allogeneic transplant. However, additional prospective studies are needed to identify the drivers of cardiovascular disease among patients receiving HSCT.

Among those undergoing allogeneic HSCT, underlying incident AF, chronic HF, CAD, CKD, cerebrovascular disease, anemia, and coagulopathy were predictors of inpatient mortality. Interestingly, dyslipidemia was noted to be associated with lower odds of inpatient mortality in the overall cohort and those undergoing allogeneic HSCT. Dyslipidemia can frequently be seen following allogeneic HSCT as a disorder of lipoprotein metabolism and is a major risk factor for CAD^22,23^. Recent evidence suggests that lipid-lowering therapy may have some role in modulating graft vs host disease and in improving cancer-associated survival in certain kinds of cancers^24^. Since the NIS does not provide data on in-hospital or outpatient pharmacologic therapies for patients, we are unable to determine whether subjects in our analysis were on lipid-lowering therapy. We suspect that a high proportion of subjects with dyslipidemia in our cohort may represent “treated dyslipidemia” and the unexpected finding of lower odds of inpatient mortality may potentially be from dyslipidemia treatment and its resultant effect on reduction in adverse cardiovascular events such as MI.

The observations are largely consistent with more isolated prior studies, which saw a higher incidence of adverse cardiovascular complications including heart failure, MI, stroke, and cardiovascular death in the long-term setting with either allogeneic or autologous HSCT alone^9^. The current study aligns with this, and shows higher risk of adverse cardiovascular events with contemporary allogeneic HSCT compared to autologous transplant. These findings are clinically significant as the use of allogeneic cell transplants continues to be expanded to wider populations. Allogeneic HSCT, as the only potentially curative treatment, is associated with lower relapse rates and better efficacy in specific populations such as those with acute myeloid leukemia, acute lymphoblastic leukemia, high-risk myelodysplastic syndrome, and multiple myeloma, however, the safety profile, particularly in the peri-HSCT setting, remains a concern as evidenced by our study^25–28^. Recognition of factors associated with adverse cardiovascular events is essential and aggressive control of cardiovascular risk factors in the peri-HSCT setting may help improve short and long-term non-relapse survival in these patients^29^. The development of predictive models such as the recently proposed CARE-BMT risk stratification score could aid in identifying (pre-transplant) high-risk patients who would benefit from timely referral to cardiovascular specialists for assessment and optimization of cardiovascular reserve prior to HSCT^30,31^. Strategies including the development and utilization of pre-HSCT risk stratification scores, identification of high-risk patients, the adoption of statin therapy to prevent treatment-induced CAD and cardiomyopathy, using cardiac biomarkers (troponin, natriuretic peptides) for early detection of cardiotoxicities, serial imaging to identify patients at risk of CAD or assessment of early left ventricular dysfunction, and the development of targeted novel strategies specific to HSCT-associated AF, such as immune-cell pathway targeted inhibition, may help further improve survival in these patients^32–36^. Additionally, there is growing data linking inflammatory pathway activation with cardiovascular outcomes, and monitoring immune activation markers (IL-1, IL-6, etc.) may have some role in long-term prognostication^37^. Prospective studies focused on identifying high-risk patients and specific management strategies to reduce cardiovascular risk are needed.

### Limitations

The results of this study should be interpreted within the context of the following limitations. First, NIS is derived from administrative billing data that relies on ICD codes which may be subject to error. However, it is worth noting that AHRQ utilizes robust quality control measures which ensure data integrity^10^. Second, we are unable to address the incidence of cardiovascular complications in a time-dependent fashion and are unable to distinguish the history of arrhythmias from new-onset arrhythmias because of the lack of distinct ICD-10 distinguishing between the incident and old arrhythmias. It is important to note that this study also focused on early (peri-HSCT) events, and could not assess incidence of cardiovascular complications in a time-dependent manner. Thus, the true long-term incidence or ramifications of AF development following HSCT could not be defined (and is likely higher). Third, we focused on in-patient data, as NIS censors data upon discharge from the facility, therefore long-term or post-discharge follow-up outcomes and subsequent AF cannot be ascertained from the database. Fourth, NIS also censors medication administration data, thus precluding the analysis of chemotherapeutic agents used (for conditioning before HSCT). Additionally, NIS does not inform upon parameters such as hospital volume or center-specific monitoring policies and clinical practices which could impact the frequency of reporting cardiac events. Given the study's retrospective nature, causation cannot be concluded, and our results merely suggest an association.

## Conclusion

In this large and nationally representative cohort of hospitalized patients undergoing HSCT, the overall prevalence of arrhythmias was high. Allogeneic HSCT was associated with worse cardiovascular outcomes and inpatient mortality, particularly among those with cardiovascular complications. These findings highlight the need for further prospective studies examining specific management strategies of cardiovascular comorbidities in the peri-HSCT setting to improve short and long-term clinical outcomes. Additional studies are needed to define the long-term incidence, mechanisms, and preventative strategies for HSCT-associated AF and other cardiovascular events.

### Supplementary Information

Supplementary Information.

## Data Availability

The datasets generated during and/or analysed during the current study are available in the [National Inpatient Sample (NIS)] repository, [https://hcup-us.ahrq.gov/nisoverview.jsp].
