# Supplementary material for: Comparison of atrial fibrillation prevalence and in-hospital cardiovascular outcomes between patients undergoing allogeneic versus autologous hematopoietic stem cell transplantation: insights from the national inpatient sample
Source: Sci Rep. 2024 Jul 22;14:16829. doi: 10.1038/s41598-024-65294-9 (PMC11263704; doi:10.1038/s41598-024-65294-9)
Supplement: Supplementary file 1 — Supplementary Information. [file 41598_2024_65294_MOESM1_ESM.docx]

**Data Supplement**

**Contents:**

1. ICD-10-CM and ICD-10-PCS codes used to identify the patient population and clinical outcomes
2. Supplemental Table 1 shows the primary diagnosis for hematopoietic stem cell transplantation (HSCT) stratified based on HSCT type.
3. Supplemental Figure 1 shows cardiovascular predictors of inpatient mortality in patients undergoing allogeneic HSCT.
4. Supplemental Figure 2 shows cardiovascular predictors of inpatient mortality in patients undergoing autologous HSCT.

ICD-10-PCS codes for allogeneic HSCT:

30243G2

30243G3

30243G4

30243U-

30243X2

30243X3

30243X4

30243Y2

30243Y3

30243Y4

30233G2

30233G3

30233G4

30233U

30233X2

30233X3

30233X4

30233Y2

30233Y3

30233Y4

ICD-10-PCS codes for autologous HSCT:

30243C0

30243G0

30243X0

30243Y0

30233C0

30233G0

30233X0

30233Y0

ICD-10-CM codes for Atrial Fibrillation:

I481-

I482-

I480

I4891

ICD-10-CM codes for Acute Kidney Injury:

N17-

ICD-10-CM codes for Cardiogenic Shock:

R570

ICD-10-CM codes for Cardiac Arrest:

I462

I468

I469

ICD-10-CM codes for Acute Heart Failure Exacerbation:

I5021

I5023

I5031

I5033

I5041

I5043

I50811

I50813

| **Supplemental Table 1: Primary diagnosis for HSCT stratified based on HSCT type** | | | |
| --- | --- | --- | --- |
|  | **Allogeneic (22,655)** | **Autologous (42,050)** | **p-value** |
| **Hodgkin Lymphoma (%)** | 340 (1.5%) | 2,625 (6.2%) | <0.0001 |
| **Non-Hodgkin Lymphoma (%)** | 1,710 (7.6%) | 9,515 (22.6%) | <0.0001 |
| **Multiple Myeloma (%)** | 410 (18.1%) | 2.3 x 10^4^ (54.9%) | <0.0001 |
| **Acute Lymphoid Leukemia** | 3,150 (13.9%) | 110 (2.6%) | <0.0001 |
| **Chronic Lymphoid Leukemia** | 245 (1.1%) | 45 (0.1%) | <0.0001 |
| **Acute Myeloid Leukemia** | 7120 (31.4%) | 200 (0.5%) | <0.0001 |
| **Chronic Myeloid Leukemia** | 630 (2.8%) | <11 | <0.0001 |
| **Aplastic Anemia (%)** | 700 (3.1%) | 65 (0.2%) | <0.0001 |
| **Chronic Myeloproliferative Neoplasm (%)** | 490 (2.2%) | <11 | <0.0001 |

For n<11, the absolute numbers are not reported as per Healthcare Cost and Utilization Project recommendations; HSCT= Hematopoietic Stem Cell Transplantation


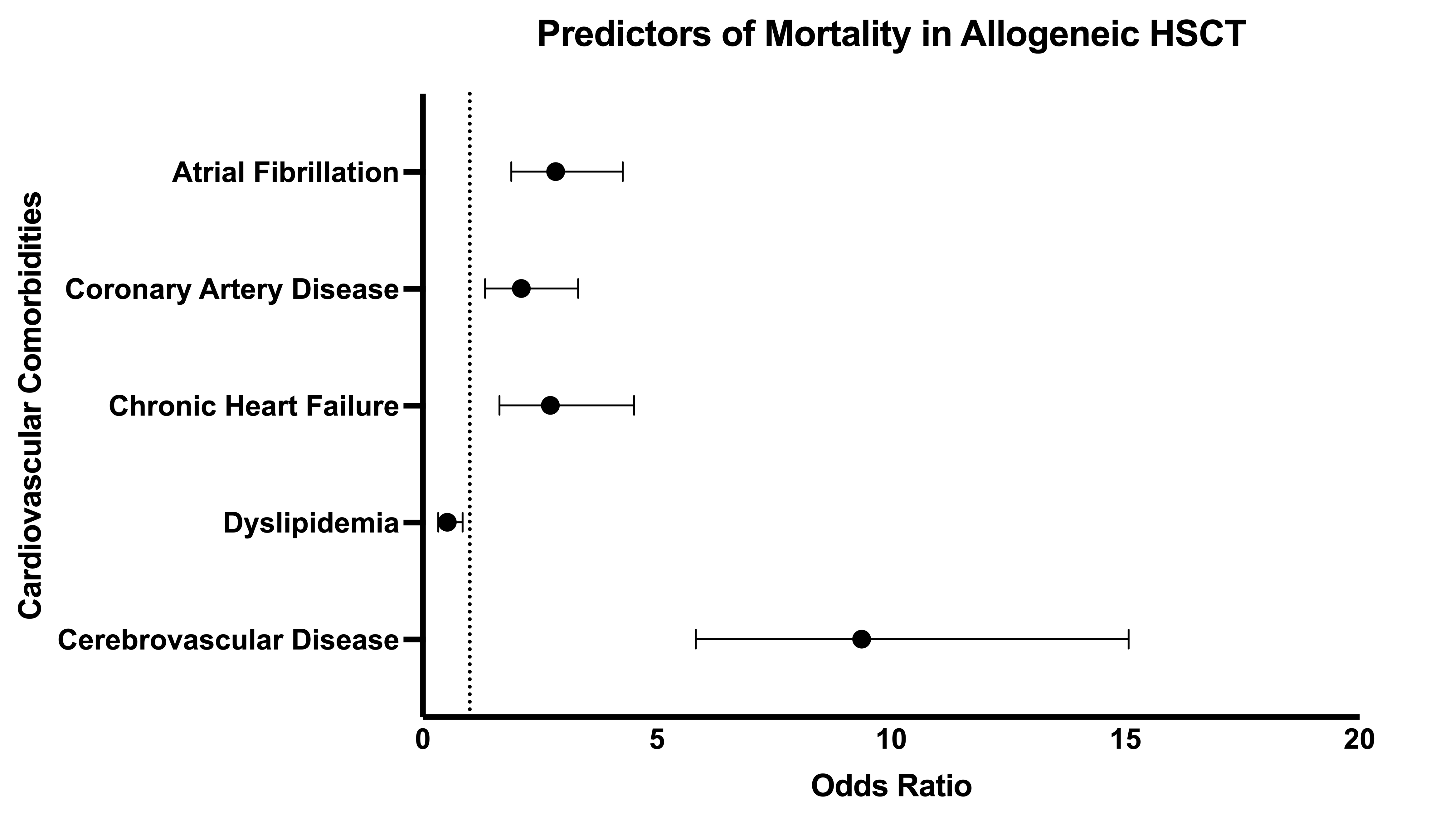


Supplemental Figure 1: Cardiovascular predictors of inpatient mortality in patients undergoing allogeneic HSCT; HSCT= Hematopoietic Stem Cell Transplantation.


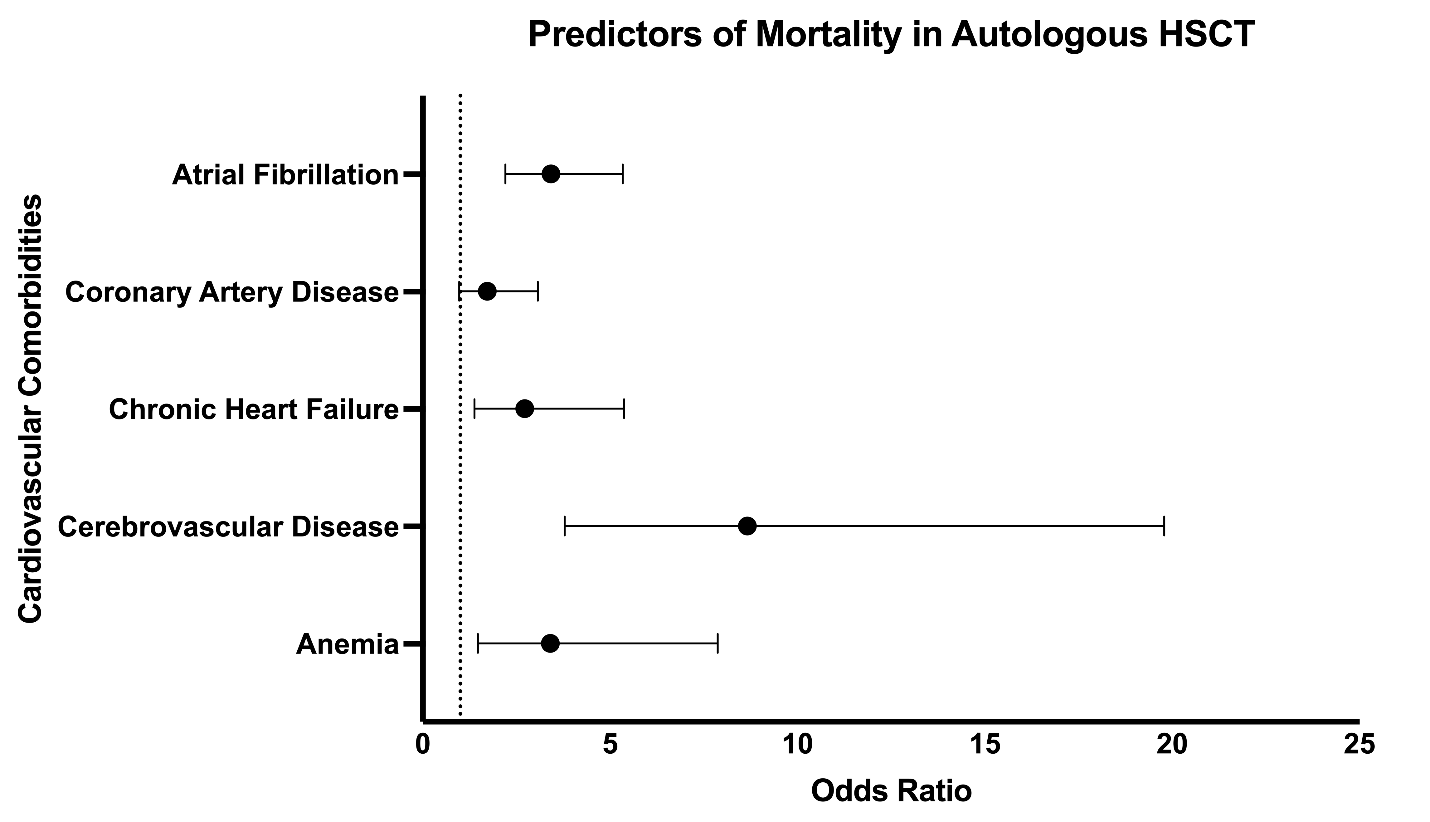


Supplemental Figure 2: Cardiovascular predictors of inpatient mortality in patients undergoing autologous HSCT; HSCT= Hematopoietic Stem Cell Transplantation.
